# L-Plastin deficiency produces increased trabecular bone due to attenuation of sealing ring formation and osteoclast dysfunction

**DOI:** 10.1038/s41413-019-0079-2

**Published:** 2020-01-22

**Authors:** Meenakshi A. Chellaiah, Megan C. Moorer, Sunipa Majumdar, Hanan Aljohani, Sharon C. Morley, Vanessa Yingling, Joseph P. Stains

**Affiliations:** 1 Department of Oncology and Diagnostic Sciences, School of Dentistry, University of Maryland, Baltimore, MD USA; 2 Department of Orthopedics, University of Maryland School of Medicine, Baltimore, MD USA; 3 Department of Pediatrics, Division of Infectious Diseases, and Department of Pathology and Immunology, Washington University School of Medicine, St. Louis, MO USA; 4 Department of Kinesiology, California State University, East Bay, Hayward, CA USA

**Keywords:** Bone, Bone quality and biomechanics

## Abstract

Bone resorption requires the formation of complex, actin-rich cytoskeletal structures. During the early phase of sealing ring formation by osteoclasts, L-plastin regulates actin-bundling to form the nascent sealing zones (NSZ). Here, we show that L-plastin knockout mice produce osteoclasts that are deficient in the formation of NSZs, are hyporesorptive, and make superficial resorption pits in vitro. Transduction of TAT-fused full-length L-plastin peptide into osteoclasts from L-plastin knockout mice rescued the formation of nascent sealing zones and sealing rings in a time-dependent manner. This response was not observed with mutated full-length L-plastin (Ser-5 and -7 to Ala-5 and -7) peptide. In contrast to the observed defect in the NSZ, L-plastin deficiency did not affect podosome formation or adhesion of osteoclasts in vitro or in vivo. Histomorphometry analyses in 8- and 12-week-old female L-plastin knockout mice demonstrated a decrease in eroded perimeters and an increase in trabecular bone density, without a change in bone formation by osteoblasts. This decrease in eroded perimeters supports that osteoclast function is attenuated in L-plastin knockouts. Micro-CT analyses confirmed a marked increase in trabecular bone mass. In conclusion, female L-plastin knockout mice had increased trabecular bone density due to impaired bone resorption by osteoclasts. L-plastin could be a potential target for therapeutic interventions to treat trabecular bone loss.

## Introduction

Osteoclasts are multinucleated, terminally differentiated giant cells, originating from the fusion of monocytes, and are involved in bone resorption. During the adhesion of osteoclasts to the bone during resorption, an actin-rich ring-like sealing zone (SZ) forms, providing a tight attachment area to the bone surface and circumscribes the area of bone resorption. The molecular dynamics of bone resorption and the cytoskeletal changes needed to carry out the formation of resorptive structures is complex and is fertile ground for therapeutic interventions to regulate bone-resorbing activities.

Sealing ring formation is a hallmark of osteoclast activation for bone resorption.^1–5^ Sealing zones are delineated as ring-shaped structures enriched in bundles of actin. Formation of actin rings defines the functionality of an osteoclast plated on mineralized matrix, dentine, or histological bone slices in vitro. In resorbing osteoclasts, rings give rise to SZs by growing individually and making a thicker and more central and stable “super-ring”.^6^ Researchers termed these structures as SZ rings, actin rings, or SZs.^7–15^ The SZ defines the resorption area of the bone, consisting of a dynamic actin-rich ring-like structure which we have designated as a sealing ring since 2007.^1,2,16–20^

Our recent work has shown an important role for plastins in osteoclast biology, and the formation of actin-rich SZ structures.^18–20^ Plastins (also known as fimbrins) are a family of three tissue-specific actin-binding proteins. Although three isoforms of plastins (L-, T-, and I-plastin) have been characterized, only L- and T-plastin are involved in cytoskeletal reorganization,^21^ and only L-plastin (LPL) can bundle β-actin efficiently.^22^ T-plastin is expressed in cells from solid tissue, whereas LPL occurs predominantly in hematopoietic cells. The third isoform, I-plastin, is specifically expressed in the small intestine, colon, and kidney.^23^ Plastins contain Ca^2+^-binding sites flanked by EF-hand motifs at the amino-terminal (NT) end and two repeated actin-binding domains (ABDs) at the C-terminal end. Each ABD contains two serial calponin-homology domains at the carboxyl-terminal end. LPL monomers bind two adjacent molecules of filamentous actin, stabilizing the parallel strands.^24^ The spatially closed ABDs (120 Å) of plastins enable them to organize actin filaments into tight bundles.^25,26^ Further, phosphorylation of LPL on Ser-5 and –Ser-7 is required for cytoskeleton rearrangements that underlie chemotaxis and adhesion.^27–29^

While LPL was reported in podosomes of osteoclasts,^30–32^ its role in osteoclastogenesis was unclear. We have previously shown colocalization of LPL and actin in the nascent sealing zones (NSZs) of resorbing osteoclasts^2^ and that phosphorylation of LPL on Ser-5 and Ser-7 regulates the actin-bundling capacity of LPL in the formation of NSZs.^18–20^

Here, we extend our earlier work and report that osteoclasts from LPL^−/−^ mice exhibit defects in the formation of sealing rings. Transduction of TAT-fused FL-LPL peptide into LPL^−/−^ osteoclasts rescued the formation of NSZs and sealing rings. This response was not observed with the mutated FL-LPL peptide at Ser-5 and Ser-7 aa. These findings strongly support a critical role of LPL phosphorylation in NSZs formation. Furthermore, LPL deficiency in mice was associated with an increase in trabecular bone volume and a decrease in eroded perimeters, indicating a mild osteopetrotic phenotype in female LPL^−/−^ mice. Analyses in LPL^−/−^ mice suggest that LPL is an essential molecule in the actin remodeling processes involved in the early phase of sealing ring formation and osteoclast function.

## Results

### LPL^−/−^ osteoclasts are not defective in osteoclast formation, podosome assembly, and migration

**Fig. 1:**
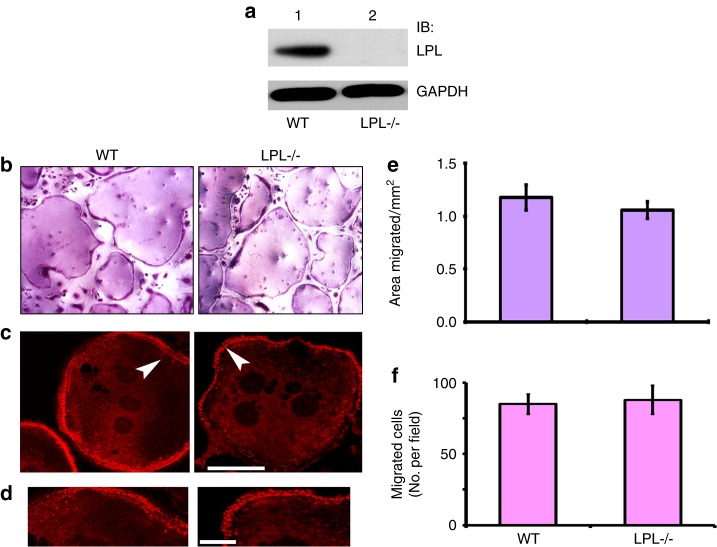
Characterization of LPL−/− osteoclasts. **a** Immunoblotting (IB) analysis with an L-plastin antibody. Equal amount of lysate proteins made from WT and LPL^−/−^ osteoclasts were used for IB with an antibody to LPL. IB with a GAPDH antibody served as a loading control. **b** Osteoclast differentiation in vitro from the bone marrow cells isolated from the long bones of WT and LPL^−/−^ mice. Representative images of TRAP-stained osteoclasts are shown. Cells were viewed under ×20 objective in a phase contrast microscope and photographed. Magnification is ×200. **c**, **d** Osteoclasts plated on coverslips were stained for actin with rhodamine-phalloidin. A magnified single osteoclast from WT (left) and LPL^−/−^ (right) mice demonstrating the dot-like podosome structures at the cell periphery is shown in **c**. Areas pointed by arrowheads in **c** are magnified in **d**. Scale bar: 100 µm in **c**; 25 µm in **d**. **e**, **f** Phagokinesis and transwell migration analyses. **e** Phagokinesis assay: the motility of the cell is represented as areas migrated in mm^2^. Results are shown as mean ± SD (*n* = 3). **f** Transwell migration assay: all assays were performed in triplicates. Experiments were repeated three times with three different osteoclast preparations. Data are presented as the number of cells per migrated field (error bars represent SD) of the three experiments performed. Data were assessed using Student’s *t* test.

An initial assessment was made in osteoclasts from wild-type control (WT) and LPL^−/−^ mice for the expression of LPL by immunoblotting analysis (Fig. 1a). As expected, osteoclasts from LPL^−/−^ mice lack LPL protein (Fig. 1a). Next, we determined the differentiation of osteoclast precursors by TRAP staining (Fig. 1b) and actin distribution in podosomes with rhodamine-phalloidin (Fig. 1c, d) in osteoclasts plated on glass coverslips. TRAP+ osteoclasts are equally formed from the bone marrow cells of LPL^−/−^ and WT mice. Actin staining with rhodamine-phalloidin demonstrated actin filament-enriched peripheral rows of dot-like structures (podosomes) in osteoclasts (Fig. 1c) from WT (left panel) or LPL^−/−^ (right panel) mice. Both phagokinesis and transwell migration assays demonstrated comparable movement in WT and LPL^−/−^ osteoclasts (Fig. 1e, f). Thus, LPL deficiency did not affect osteoclast adhesion and migration.

### LPL^−/−^ osteoclasts are defective in NSZ formation and resorption pit formation on dentine matrix

**Fig. 2:**
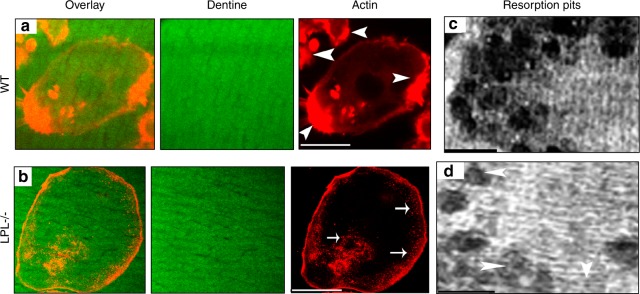
Analysis of the formation of nascent sealing zones (NSZs) and dentine resorption activity in WT and LPL−/− osteoclasts. Osteoclasts from WT (**a**) and LPL^−/−^ (**b**) mice were cultured on dentine slices for 3 h–4 h in the presence of TNF-α and stained for actin with rhodamine-phalloidin. Confocal microscopy analysis was done in actin-stained osteoclasts. Dentine is shown in green color (pseudocolor) and actin in red. Overlay image shows the distribution of actin (red) on a dentine slice (green). Arrowheads point to NSZs in WT osteoclasts and arrows point to podosomes in LPL^−/−^ osteoclasts. Scale bar—50 µm. Experiments were repeated three times in osteoclasts isolated from WT and LPL^−/−^ mice. The number of NSZs were counted in ~60 WT osteoclasts total from three different experiments and the average number of NSZs is ~146 ± 21 (mean ± SD). **c**, **d** Analysis of the resorption activity in osteoclasts plated on dentine slices. Osteoclasts were cultured on dentine slices for 10 h–12 h in the presence of TNF-α. Resorption pits were scanned using a Bio-Rad confocal microscopy. Resorbed area is seen as dark areas. Arrowheads in **d** point to superficial pits. Scale bar—25 µm.

Prior work has implicated LPL as a component of podosomes, which are used by macrophages to support migration, polarization, and lamellipodia formation.^33–38^ Accordingly, we examined if the loss of LPL affects podosome formation in osteoclasts. Regardless of genotype, osteoclasts plated on glass coverslips demonstrated actin-rich dot-like podosomes at the cell periphery (Figs. S1A and 2). Confocal microscopy analyses of the osteoclasts plated on dentine slices consistently revealed NSZs at 3 h–4 h (Fig. S1B), and mature sealing rings at 10 h–12 h (Fig. S1C).^2,18,19^ To further elucidate the possible role of LPL in NSZs formation, we used osteoclasts from WT and LPL^−/−^ mice. Osteoclasts from WT mice demonstrated NSZs (Fig. 2a; indicated by arrowheads) and multiple over-lapping resorption pits (Fig. 2c) when plated on dentine matrix. NSZs were counted in ~60 osteoclasts from three different experiments (~20/experiment), and the average number of NSZ is ~146 ± 21 (~2.4 NSZ per osteoclast). In contrast, LPL^−/−^ osteoclasts were defective in the formation of NSZs (Fig. 2b). As a result, these osteoclasts were hyporesorptive and made superficial pits in vitro (Fig. 2d).

**Fig. 3:**
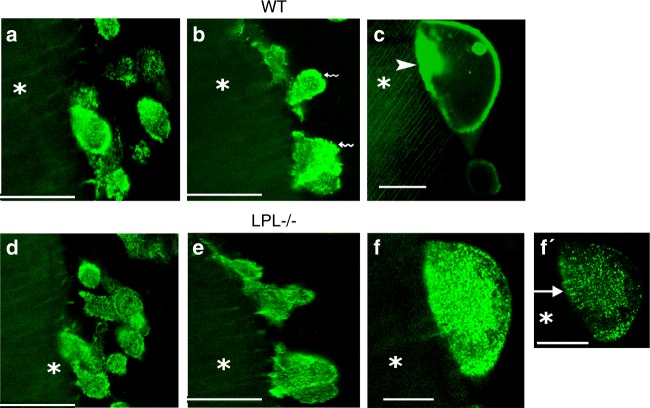
Intermittent or short time-lapse video analyses in osteoclasts from wildtype (WT) and L-plastin knockout (LPL−/−) mice. Short time-lapse video microscopy analyses at 45–60 min (**a**, **d**) 2 h–3 h (**b**, **e**), and 3 h–4 h (**c**, **f**, **f′**) in WT (**a**–**c**) and LPL^−/−^ osteoclasts (**d**–**f**) expressing GFP-actin are shown. Osteoclasts were incubated with dentine slices and TNF-α during these analyses. Basolateral membrane-like structures are indicated by wavy arrows in **b**. NSZ is indicated by an arrowhead in **c**. Podosome-like structures are indicated by an arrow in **f′**. The asterisk in **a**–**f′** indicates dentine matrix which is shown in diffused green color (pseudocolor). Scale bars—100 μm (**a**, **b**, **d**, **e**); 50 μm (**c**, **f**, **f′**).

To further validate the observations shown above, we performed intermittent time-lapse live video analyses at 45–60 min (Fig. 3a, d), 2 h–3 h (Fig. 3b, e), and 3 h–4 h (Fig. 3c, f) in GFP-actin expressing WT (Fig. 3a–c) and LPL^−/−^ osteoclasts (Fig. 3d–f). Osteoclasts from WT and LPL^−/−^ mice actively migrate towards dentine slices at 45–60 min. Irrespective of genotype, these osteoclasts demonstrate podosome-like structures (Fig. 3a, d) and attach to dentine slices at 2 h–3 h (Fig. 3b, e). Organization of actin-rich basolateral membrane occurs opposite to the attachment zone to dentine in WT osteoclasts (Fig. 3b, indicated by wavy arrows). However, actin distribution is punctate in the membrane of LPL^−/−^ osteoclasts (Fig. 3e). At 3 h–4 h, the WT osteoclasts form actin aggregate-like structures or NSZs (Fig. 3c, indicated by an arrowhead). Contrastingly, LPL^−/−^ osteoclasts fail to form NSZs (Fig. 3f, f′). An arrow in f′ points to podosome-like structures which support the adhesion of LPL^−/−^ osteoclasts to dentine surface.

### Transduction of a TAT-fused full-length LPL peptide rescues the formation of sealing rings in LPL^−/−^ osteoclasts

**Fig. 4:**
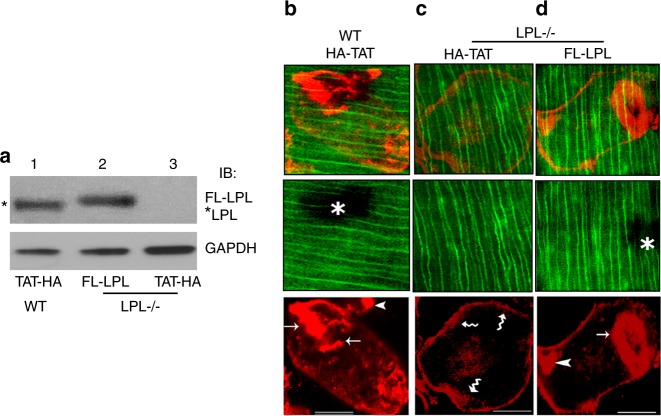
Analysis of the effect of transduction of indicated TAT-fused peptides on the formation of sealing rings in osteoclasts from wildtype (WT) and L-plastin knockout (LPL−/−) mice. **a** Immunoblotting analysis with an antibodyto LPL. WT and LPL^−/−^ osteoclasts treated with bone particles and TNF-α were transduced with indicated TAT-fused FL-LPL (lane 2) or TAT-HA (lanes 1 and 3) peptide. Immunoblotting analysis with an antibody to LPL demonstrated endogenous LPL protein in WT osteoclasts (~68–70 kDa; indicated by asterisks) and transduced FL-LPL peptide in LPL^−/−^ osteoclasts (~75–78 kDa; lane 2). Immunoblotting with a GAPDH antibody was used as loading control. **b**–**i** Osteoclasts transduced with indicated TAT-fused peptides were plated on dentine slices for 14 h–16 h in the presence of TNF-α to determine the formation of sealing rings (**b**–**d**). Confocal images of osteoclasts are shown. Merged (red and green) images are shown in the top panels. Dentine is shown in green color (pseudocolor; middle panels). An asterisk in **b** and **d** points to resorbed area underneath the osteoclast. Actin-stained cells are shown in the bottom panels. Arrows point to sealing rings and arrowheads point to NSZs. Wavy arrows point to podosomes. Scale bar—50 μm. Experiments were repeated three times with three different osteoclast preparations.

We have previously shown that transduction of TAT-fused full-length LPL (FL-LPL) peptide significantly increased the number of NSZs and therefore, sealing rings in WT osteoclasts.^18^ Here, we examined if the transduction of TAT-fused FL-LPL could rescue the defect in the formation of sealing rings in LPL^−/−^ osteoclasts. Accordingly, LPL^−/−^ osteoclasts were transduced with the TAT-fused FL-LPL or TAT-HA vector control peptide. TAT-HA peptide transduced WT osteoclasts were used as controls. Immunoblotting analysis with an LPL antibody confirmed the transduced levels of FL-LPL peptide (~75–78 kDa, Fig. 4a, lane 2) in LPL^−/−^ osteoclasts. FL-LPL is adequately transduced in LPL^−/−^ osteoclasts, and it is comparable with the levels of endogenous LPL protein (~68–70 kDa; lane 1) in WT osteoclasts transduced with the vector peptide TAT-HA. Consistently, LPL^−/−^ osteoclasts are lacking LPL protein (lane 3).

Osteoclasts (WT and LPL^−/−^) transduced with indicated TAT-peptides were plated on dentine slices for 10 h–12 h in the presence of TNF-α. Low dose TNF-α promotes actin ring formation in osteoclasts, which is associated with bone loss that occurs in inflammatory diseases.^39–42^ LPL^−/−^ osteoclasts transduced with TAT-HA peptide demonstrated podosome-like structures at the periphery but lacked well-formed sealing rings (Fig. 4c). Transduction of LPL^−/−^ osteoclasts with the TAT-fused FL-LPL peptide rescued sealing ring formation (Figs. 4d and S2). Pit forming activity corresponds with the rescue of the sealing ring formation in FL-LPL transduced LPL^−/−^ osteoclasts (Fig. S3C, D). In contrast, neither sealing rings nor resorption pits were observed in LPL^−/−^ osteoclasts transduced with TAT-HA peptide (Figs. 4c, S2 and S3).

**Fig. 5:**
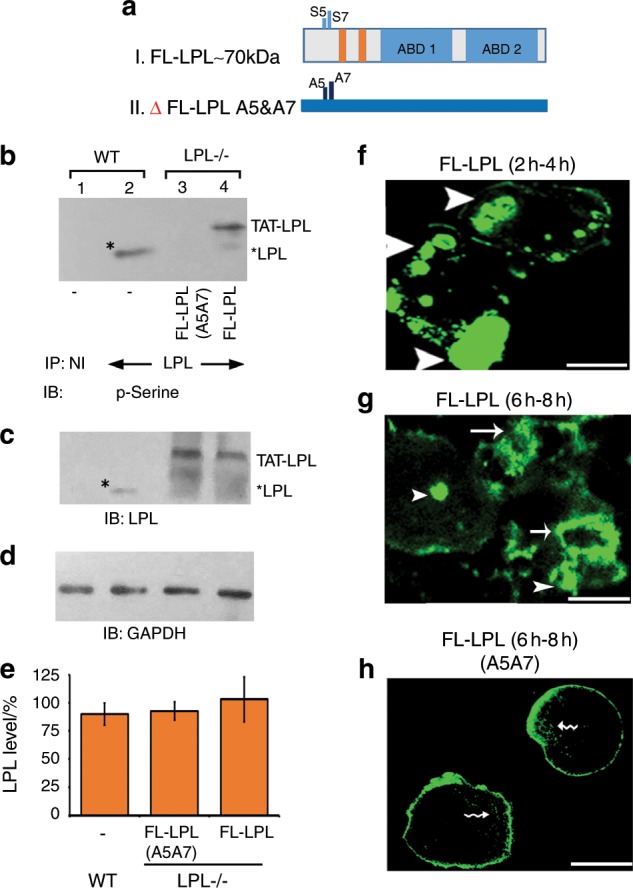
Intermittent time-lapse video microscopy analyses in LPL−/− OCs transduced with TAT-fused FL-LPL peptides. **a** Schematic diagram demonstrating the structure of FL-LPL (I) and mutated (Δ) FL-LPL (A5A7) (II) peptides. **b**–**e** Immunoblotting (IB) analysis: immunoprecipitates made with an antibody to LPL (lanes 2–3) or nonimmune serum (NI; lane 1) were immunoblotted with an antibody to phosphoserine (p-Serine; **b**). Stripping and reprobing with an LPL antibody is shown in **c**. Asterisks indicate endogenous LPL in WT osteoclasts (**b**, **c**). Equal amount of lysate protein (Input) used for the immunoprecipitation was assessed by direct IB of the lysates with a GAPDH antibody (**d**). Transduced protein levels from three different experiments are shown as a graph in **e** (error bar represents SD). Data were assessed by standard Student’s *t* test. There is no statistically significant difference in the levels of LPL protein between groups. **f**–**h** Osteoclasts expressing GFP-actin were transduced with TAT-fused FL-LPL (**f**, **g**) or FL-LPL (A5A7; **h**) for 15 min; then plated on dentine slices and supplemented with TNF-α. Analyses were done at 2 h–4 h (**f**) and 6 h–8 h (**g**, **h**). NSZs are indicated by arrowheads in **f** and **g**. Single and multiple sealing rings (indicated by arrows) are observed at 6 h–8 h in LPL^−/−^ osteoclasts transduced with FL-LPL (**g**). Actin distribution was observed in podosomes (indicated by wavy arrows) and the plasma membrane in LPL^−/−^ osteoclasts transduced with ΔFL-LPL (A5A7). The results shown are representative of three different experiments from three different osteoclasts preparations. Scale bar—25 μm.

**Fig. 6:**
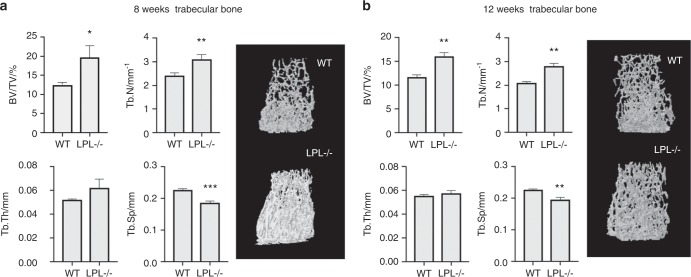
Micro-CT analysis of the trabecular compartment of wild-type and LPL−/− mice. Trabecular bone microarchitecture was assessed by micro-CT at the distal femoral metaphysis of (**a**) 8- or (**b**) 12-week-old wild-type or LPL^−/−^ female mice by micro-CT (*n* = 5–6 mice per group). Trabecular bone volume fraction (BV/TV), trabecular number (Tb.N), trabecular thickness (Tb.Th), and trabecular spacing (Tb.Sp) are shown. Data are mean ± SD. *P < 0.05; **P < 0.01; ***P < 0.001 vs. WT mice. Statistical analysis was performed using Student's *t* test. A representative image of the trabecular bone in the ROI is shown for each genotype.

**Fig. 7:**
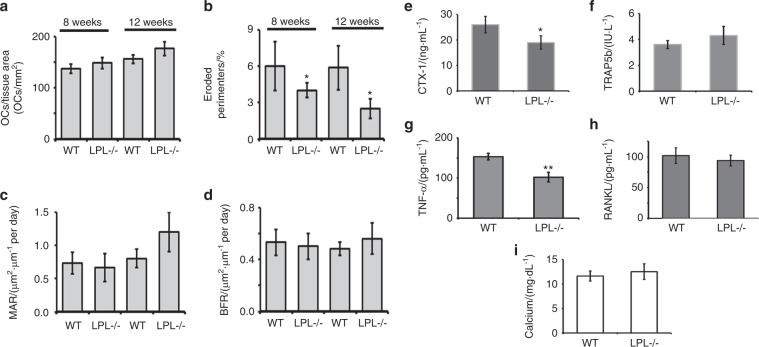
Comparison of the histomorphometric parameters and factors that regulates bone modeling in WT and LPL−/− mice. **a**–**d** Histomorphometric assessments of indicated bone parameters are provided as bar graphs. Other parameters are provided in the Supplementary Fig. S7 and Table S1. Analyses were done twice. Each time 5–7 mice were used for each group. Data shown are mean ± SEM; *P < 0.05; **P < 0.01 vs. WT mice. **e**–**i** Comparison of serum levels of indicated biomarkers of bone formation and resorption in WT and LPL^−/−^ mice. CTX-1 (**e**), TRAP (**f**), TNF-α (**g**), RANKL (**h**), and Calcium (**i**), were measured by ELISA in the serum of 12-week-old WT and LPL^−/−^ mice. The data represent mean ± SEM of 5–6 mice per group (*n* = 2). *P < 0.05; **P < 0.01 vs. mice. *P* values were assessed by standard Student’s *t* test.

The ability of TAT-fused FL-LPL peptide to modulate actin dynamics in osteoclasts requires phosphorylation at Ser-5 and Ser-7.^18^ Here, we sought to determine whether the transduced FL-LPL peptide is phosphorylated and if the phosphorylation influences the formation of NSZs. Therefore, LPL^−/−^ osteoclasts were transduced with TAT-fused FL-LPL or mutated FL-LPL (A5A7) peptide (schematic diagram in Fig. 5a). Subsequently, WT (control) and transduced LPL^−/−^ osteoclasts were treated with bone particles and TNF-α for 3 h–4 h to make lysates for immunoprecipitation (Fig. 5b–d). LPL immunoprecipitates were first immunoblotted with a p-Serine antibody (Fig. 5b). Lysates made from WT osteoclasts were used as a reference standard for LPL phosphorylation (lane 2). Phosphorylation of endogenous LPL in WT osteoclasts (lane 2) and the transduced FL-LPL (lane 4) peptide was observed. Although phosphorylation of the transduced mutated FL-LPL was not observed (panel b; lane 3), immunoblotting with an LPL antibody demonstrates the transduced mutated and unmutated FL-LPL peptides (panel c; lanes 3 and 4) and the endogenous LPL peptide in WT osteoclasts (panel c; lane 2). Consistent with the observations shown in Fig. 2, the molecular mass of transduced FL-LPL is ~75–78 kDa in LPL^−/−^ osteoclasts and endogenous LPL protein is ~68–70 kDa in WT osteoclasts. Densitometric quantification of the transduced and endogenous protein levels from three different experiments are shown as a graph in panel e. There is no statistically significant difference in the levels of LPL protein between groups (Fig. 5e). We then determined the time-dependent organization of NSZs and sealing rings using intermittent time-lapse video recording (Fig. 5f–h). After transduction, LPL^−/−^ osteoclasts were plated on dentine slices in the presence of TNF-α. At the specified time of analysis (2 h–4 h in Fig. 5f and 6 h–8 h in Fig. 5g), time-lapse video recording was performed every 15′ for 2 h. Representative frames are shown at 3½ h (Fig. 5f) and 7 h (Fig. 5g, h). As anticipated, transduction of the TAT-fused FL-LPL peptide rescued the formation of NSZs and sealing rings in a time-dependent manner in LPL^−/−^ osteoclasts (Fig. 5f, g). This rescue was not observed with FL-LPL (A5A7) (panel h), supporting the notion that these phosphorylation sites are essential to LPL’s action in the formation of NSZs and sealing rings. These osteoclasts demonstrated podosome-like structures (indicated by wavy arrows (panel h)). However, fewer podosome-like structures were observed on dentine than on glass coverslips (Figs. 2 and 5), which may be due to the effects of different substratum.^6,43–45^ Time-lapse analyses in three different experiments validating the effects TAT-fused FL-LPL and FL-LPL (A5A7) peptides on the rescue of NSZs and sealing rings are shown in quadruplicates (Fig. S4). The above results confirm an essential role for LPL in the ability of the osteoclasts to form the structures necessary to resorb bone efficiently.

### LPL deficiency increased trabecular bone in femurs of 8- and 12-week-old female mice

Homozygous LPL^−/−^ mice develop normally and are fertile. At 8 and 12 weeks of age, female LPL^−/−^ mice did not show any anomaly in body length or weight as well as in the histology of organs such as liver, brain, kidney, and heart compared with WT mice. Femurs of 8- and 12-week-old mice were analyzed using micro-CT. Also, tibias and femurs of 8- and 12-week-old mice were subjected to quantitative histology and histomorphometry analyses.

Consistent with prior work,^46^ micro-CT analyses demonstrated a marked increase in trabecular bone mass (BV/TV) in the distal femur of LPL^−/−^ mice at both 8 weeks (Fig. 6a) and 12 weeks of age (Fig. 6b). This is associated with an increase in trabecular number (Tb.N) and decrease in trabecular separation (Tb.Sp). In contrast, at 8 weeks of age, there was no significant difference in cortical parameters at the femoral mid-diaphysis (Fig. S5, panels in a). However, by 12 weeks of age, a decrease in cortical bone thickness (Cs.Th) was observed in LPL^−/−^ mice relative to their WT controls (Fig. S5). This decrease in Cs.Th was likely not due to increased periosteal apposition, as the periosteal perimeter (Ps.Pm) of the mid-diaphysis did not differ between genotypes (Fig. S5B, middle panel). Instead, there was a significant increase in the endocortical perimeter (Ec.Pm), suggesting an increase in endocortical bone resorption in these mice (Fig. S5B, right panel).

Static bone histomorphometric analysis was performed in bone sections (femur and tibia) stained with hematoxylin and eosin (H&E) staining (Fig. S6A, C). Bone sections were also stained for TRAP to aid in the detection of osteoclasts (Fig. S6B, D). Consistent with the micro-CT analyses, histomorphometric analyses also demonstrated an increase in trabecular bone density in LPL^−/−^ mice by 8 weeks of age (Fig. S6C, D; Table S1). The number of TRAP-positive osteoclasts present on the surface of WT and LPL^−/−^ bones were similar (Fig. 7a; Table S1). Despite these similar numbers of osteoclasts, LPL^−/−^ mice show (a) a decrease in the eroded perimeter (Fig. 7b), suggesting a decrease in osteoclast activity; and (b) a significant increase in the trabecular thickness and Tb.N corresponding with a decrease in the trabecular spacing (Fig. S6F–H; Table S1). Both of these results are consistent with a decrease in osteoclast activity in both 8- and 12-week-old female mice when LPL is not present (Fig. 7b).

In order to assess the contribution of osteoblast-mediated bone formation to this phenotype, mice were injected with calcein at 2 and 7 days before sacrificing.^47,48^ Dynamic histomorphometry showed no difference in bone formation rate or the mean mineral apposition rate (Fig. 7c, d) between genotypes. Likewise, the number of osteoblasts is not different in bones from LPL^−/−^ and WT mice (Table S1). These data support the notion that the increase in trabecular bone density is not due to osteoblast-mediated bone formation.

### LPL deficiency had differential effects on the serum levels of cytokines, calcium, and bone resorption markers

To confirm that LPL deficiency in mice blocked osteoclast bone resorption and not osteoclast differentiation, we examined the biomarkers of bone resorption in the serum of LPL^−/−^ mice. Similarly, bone formation biomarkers and calcium levels were also measured. Serum from WT mice was used as a control. Consistent with defects in osteoclast activity, C-terminal telopeptide of type 1 collagen (CTX) levels were significantly lower in LPL^−/−^ mice as assessed by ELISA (Fig. 7e). Consistent with a similar number of osteoclasts present between both genotypes, the levels of TRAP, RANKL, and calcium remained the same between genotypes (Fig. 7f, h, and i). A decrease in eroded perimeters prompted us to determine the serum levels of TNF-α. Serum TNF-α is decreased in LPL^−/−^ compared with WT mice. TNF-α is capable of causing bone erosion either by enhancing proliferation or activity of cells in the osteoclast lineage.^40,49^ A decrease in the eroded perimeter (Fig. 7b) suggests that a decrease in circulating TNF-α may diminish the overall function of osteoclasts.

### LPL deficiency did not affect the biomechanical properties of the bone

Here, we used femurs and tibia isolated from WT and LPL^−/−^ mice at 13 weeks of age to evaluate the bone strength when LPL is not present. However, no significant differences were found in the biomechanical properties of either the femur or the tibia between groups (Table S2; Fig. S7). This was surprising three-point bending mostly measures the biomechanical properties of the cortical bone, which micro-CT analyses revealed a significant decrease in Cs.Th (Fig. S5; left panel in b). Perhaps this was due to the increased bone mass in the trabecular compartment of LPL^−/−^ mice, as trabecular bone in the diaphyseal area may also contribute to total bone strength.

## Discussion

Regulation of sealing ring formation during bone resorption by osteoclasts is critical in pathological bone loss. This study was designed to demonstrate the essential role of LPL in osteoclast actin dynamics using LPL^−/−^ mice. Consistent with prior work,^2,18,19^ we showed that LPL, an actin-bundling protein, plays a vital role in the formation of NSZs, which mature into fully functional sealing rings. LPL deficiency results in a mild osteopetrosis phenotype with increased trabecular bone volume and a decreased in eroded perimeters. These are consistent with the possible deficiency in the actin modeling processes involved in the formation of sealing rings in LPL^−/−^ osteoclasts. LPL deficiency did not affect osteoclast differentiation in vitro. Likewise, bone histomorphometry analysis showed that the number of osteoclasts is not significantly different between WT and LPL^−/−^ mice. LPL deficiency did not affect the adhesion of osteoclasts to the bone surface due to the presence of podosomes. Rather, the skeletal phenotype in LPL^−/−^ mice is likely due to the inhibition of bone resorption. Decreased serum levels of CTX-1, a bone resorption marker, validate this observation of reduced bone resorption.

Podosomes are the primary adhesive structure of the osteoclasts.^47,50–54^ LPL is indispensable for podosome formation, stability, and function in macrophages.^38,55^ However, LPL^−/−^ osteoclasts are well spread on coverslips, and actin staining revealed distinct podosome structures in the clear zone area. Consistent with functional podosomes, migration is not affected in LPL^−/−^ osteoclasts. Overall, these results suggest that the mechanism of formation of podosomes is cell-type dependent.

In a previous study with Gsn^−/−^ osteoclasts, we showed that Gsn deficiency blocks podosome assembly and motility. However, these osteoclasts still exhibited sealing rings and matrix resorption. Therefore, Gsn^−/−^ osteoclasts are capable of resorbing bone, but the resorbed areas are small as a result of the absence of podosomes and the hypomotile nature of Gsn^−/−^ osteoclasts.^47^ Previous studies have shown that podosomes change their dynamic property when they mature into belts or actin rings in a SZ area. When doing so, a major change in the actin filament assembly takes place in the formation of SZs. SZs are made of structural units related to individual podosomes with more extended actin core and higher density of inter-connecting actin filaments.^56–58^ The dynamic reorganization of actin filaments is regulated by the external milieu of osteoclasts.^43,59^ Thus, we examined sealing ring formation in osteoclasts plated on dentine slices, on which the mineralized matrix (organic and inorganic component), its solubility, and the surface area of the crystals impact the resorption kinetics and the architecture of the resorption pits.^59^ Observations in Gsn^−/−^ and LPL^−/−^ osteoclasts suggest that podosomes and sealing rings may have unique actin regulatory mechanisms although they display comparable molecular composition.

LPL phosphorylation is regulated by signaling pathways consisting of different kinases including PKC, PKA, Src, etc.^21,46,60–64^ The results presented here validate our previous studies^2,18–20^ that LPL phosphorylation is essential in the regulation of NSZ formation by TNF-α signaling. A limitation of our study is that we did not explore the mechanisms regulating LPL phosphorylation. Recent studies have begun to focus on the TNF-α mediated signaling mechanism(s) involved in the phosphorylation of LPL in osteoclasts.

Micro-CT and histomorphometric analyses showed an increase in trabecular bone mass consistent with osteopetrosis. Even though a decrease in Cs.Th was observed in 12-week-old female LPL^−/−^ mice; there was no significant change in periosteal measures and no difference in mechanical strength at the mid cortical sites in both the femur and tibia of female mice. As suggested by others, there are several likely possibilities for how bone resorption can be attenuated without unfavorable reduction or increase in bone formation,^65,66^ e.g., human osteopetrosis, caused by mutations in proteins involved in the acidification of the resorption lacuna (Chloride channel ClC-7 or the a3-V-ATPase). This mutation decreased resorption with normal or even increased bone formation.^65^ The defect in osteoclast function did not affect endochondral bone growth in mice analyzed at 8 and 12 weeks, nor did shortening, and clubbing of the femurs occur as reported in some of the osteopetrotic mice.^67^ Despite changes in trabecular microarchitecture and osteoclast activity, serum calcium levels remain the same in LPL^−/−^ and WT mice. Regardless, these data suggest that LPL might have a negative role in the cortical compartment and whether this role changes with increasing age will be the focus of future studies.

To conclude, osteoclasts from LPL^−/−^ osteoclasts failed to form NSZs; however, these osteoclasts demonstrated actin-enriched peripheral podosome-like structures. Deletion of LPL is associated with an increase in trabecular bone volume and a decrease in eroded perimeters, indicating osteopetrosis, but no deficits in bone strength at mid-diaphyseal cortical sites. LPL deficiency did not affect or increase bone formation by osteoblasts. Our study suggests that LPL deficiency causes abnormalities in osteoclast function appear to move in opposite directions in the trabecular and endocortical regions of long bones. LPL-dependent and -independent mechanisms of actin assembly occur at the metaphyseal and endocortical regions of long bones, respectively. LPL-independent assembly mechanisms appear to be capable of founding sealing rings for bone resorption to occur in the endocortical region. In the future, it is crucial to identify whether there is any compensatory mechanism that could be present in this region for the observed bone loss. LPL seems to act primarily at trabecular sites to regulate osteoclastic resorption, suggesting that LPL is a novel target for therapeutic intervention to block bone loss. However, further analyses are required to determine how osteoclasts deficient in LPL expression at endosteal areas could perform differently from those at the trabecular surfaces.

## Materials and methods

### Materials

Antibody to LPL (SC-16657; Goat) was purchased from Santa Cruz Biotechnology (Santa Cruz, CA). Antibodies to GAPDH and TNF-α receptor 1 (TNFR1) were purchased from R & D Systems (Minneapolis, MN). Protein estimation reagent, molecular weight standards, and PAGE reagents were bought from Bio-Rad (Hercules, CA). Cy2- and Cy3-conjugated secondary antibodies were purchased from Jackson Immunoresearch (West Grove, PA). HRP-conjugated secondary antibodies for immunoblotting and the phosphoserine (p-Serine) antibody were purchased from Abcam (Cambridge, MA). Mounting solutions for mounting of coverslips were purchased from Thomas Scientific (Swedesboro, NJ) or Vector Labs (Burlingame, CA). Rhodamine-phalloidin and all other chemicals were purchased from Sigma (St. Louis, MO).

### Mice

C57/BL6 mice (6–8-week-old mice) were used for osteoclast preparation. These mice were either purchased from Harlan Laboratories or generated in the animal facility of the University of Maryland Dental School. LPL^−/−^ mice colony established initially at Washington University by homologous recombination at the LPL gene in ES cells were used.^27^ Mice were back-crossed in to generate LPL^−/−^ mice on a C57Bl6 background.^35^ The analyses described in this paper were done in LPL^−/−^ and WT mice on a C57Bl6 background. Breeding and maintenance were carried out as per the guidelines and approval of the University of Maryland institutional animal care and use committee (IACUC).

### Preparation of osteoclasts from long bones

Osteoclasts were generated in vitro using long bone marrow cells of 6–8-week-old C57BL/6 mice as described previously.^16,47^ The multinucleated osteoclasts were seen from day 4 onward. Mature osteoclasts were replated on dentine for immunostaining and bone resorption analyses.

### Lysate preparation and immunoblotting analysis

Osteoclasts generated from WT and LPL^−/−^ bone marrow were washed three times with cold PBS and lysed in RIPA buffer (RIPA; 10 mmol·L^−1^ Tris-HCl, pH 7.2, 150 mmol·L^−1^ NaCl, 1% deoxycholate, 1% Triton X-100, 0.1% SDS, 1% aprotinin, 2 mmol·L^−1^ PMSF, 100 mol·L^−1^ Na_3_VO_4_, and 1% aprotinin). An equal amount of lysate proteins (50 µg) made from WT and LPL^−/−^ osteoclasts were used for immunoprecipitation or immunoblotting analyses.^2,18^

### Expression of GFP-actin in osteoclasts and confocal microscopy analysis of the time-dependent formation of NSZs and sealing rings in live cells

Osteoclasts were transfected with human ß-actin containing an NH2-terminal EGFP vector (Clontech) using the transfection reagent (MIRUS). Expression of GFP-actin was confirmed at 10 h–12 h after transfection. Dentine slices were added to osteoclast cultures and images were acquired at 45–60 min, 2 h–3 h and 3 h–4 h in the presence of TNF-α in WT and LPL^−/−^ osteoclasts with a 1.4 NA Plan Apo ×63 objective (Bio-Rad confocal microscopy). GFP-actin expressing cells transduced with TAT-fused peptides (mutated and unmutated TAT-fused FL-LPL) were also plated on dentine slices, and intermittent time-lapse analyses were done at 2 h–4 h and 6 h–8 h in the presence of TNF-α.

### Transduction of TAT-fused peptides into osteoclasts

We used pTAT-HA expression vector^68^ for cloning and purification of the FL-LPL and mutated FL-LPL (Ser-5 and Ser-7 substituted to Ala-5, and Ala-7; abbreviated as FL-LPL (A5A7)) constructs. Mutations at Ser-5 and Ser-7 were generated using the Quick-Change Site-Directed Mutagenesis Kit (Agilent Technologies, Halethorpe), as described.^18^ FL-LPL cDNA (Accession: BC010271) were used to generate the mutant.^2^ Purification of TAT-fused peptides and transduction into osteoclasts were completed as previously described.^69^

### Fluorescent labeling of proteins in osteoclasts

Osteoclast precursors (10^5^ cells/coverslips) were cultured on glass coverslips or dentine slices. Fluorescent labeling was done with rhodamine-phalloidin to determine actin organization.^47,69^ Immunostained and actin-stained osteoclasts were imaged using a Bio-Rad confocal laser-scanning microscope. Images were stored in TIF image format and processed by Adobe Photoshop (Adobe Systems Inc., Mountain View, CA).

### Migration assays

Transwell and phagokinesis migration assays were done as previously reported.^47,69,70^ Statistical significance was calculated as described below. In phagokinesis assay, the migration efficiency was evaluated by measuring the areas free (tracks) of gold particles. A gridded reticle (Boyce Scientific, Inc., Gray Summit, NC) was used in the eyepiece of a Nikon microscope and tracks were measured using a ×10 objective. Each assay was done in triplicates with WT and LPL^−/−^ osteoclasts, and migratory tracks were measured in 10–15 cells/assay; assays were repeated three times with three different osteoclast preparations. An average of 30–40 tracks is provided as area moved in mm^2^.

After migration in transwell chambers for 12 h–14 h, remaining cells that did not migrate from the upper side of the transwell were removed gently with a cotton-tipped applicator. Membranes were then stained with hematoxylin stain (Sigma) after fixing the migrated cells with an alcohol/formaldehyde/acetic acid mixture (20:2:1) for 15 min. Membranes were rinsed well with water and dried. Dried filters were mounted on a glass slide and counted in an inverted microscope (Zeiss microscope) as previously described.^69^ About 5–6 areas from each membrane were counted. All assays were done in triplicates. Therefore, the average of 15–18 picture fields/experiment at ×100 magnification was quantified. Assays were repeated three times with three different osteoclast preparations. The number of fields was >45 in each assay. Data presented are mean ± SD of three experiments in both assays.

### Dentine matrix resorption assay in vitro and measurement of the pit area

Osteoclasts were replated on dentine slices for 10 h–12 h in the presence of TNF-α. Resorbed areas were scanned using Bio-Rad confocal microscopy essentially as previously described.^47^ The dentine slices mounted on No. 1 coverslips were viewed with a 40 × 0.6 NA air objective. Images were recorded in the epi-reflection mode using the 488-nm line of the argon laser in a 512 × 512-pixel format. Data collection and processing were accomplished with the Zeiss LSM 410 software package. Photomicrographs were stored in a TIFF image format. The area of the pit was determined from the free-hand traced perimeter using the LSM software Area Measurement function. Images were stored in TIF format and processed by Adobe Photoshop (Adobe Systems Inc.).^47^

### Microcomputed tomography

Femurs were dissected from 8- to 12-week-old female WT and LPL^−/−^ mice and processed as described.^71^ Three-dimensional Micro-CT was performed on femurs (*n* = 5–6) using a SkyScan 1172 (Bruker, Kontich, Belgium) at 60 kV (167 µA) and a 9.91 µm voxel size, as described.^72,73^ The skeletal parameters assessed by micro-CT followed published nomenclature guidelines.^74^ Trabecular bone microarchitecture, including the trabecular bone volume fraction (BV/TV), trabecular bone thickness (Tb.Th), Tb.N, and Tb.Sp, was analyzed in a manually delineated region of interest 0.25 mm–2.5 mm proximal to the distal femoral growth plate. Cortical parameters, including Cs.Th, periosteal (Ps.Pm), and endocortical or endosteal perimeters (Ec.Pm), were 0.6 mm region at the femoral mid-diaphysis.

### Bone histomorphometry

Bone histomorphometry was done in 8-week-old female LPL^−/−^ and WT mice as described.^47,48^ Static and dynamic histomorphometric measurements were made using Bio-Quant software. All measurements were done to the metaphyseal region distal to the growth plate region. To estimate bone formation rate, double-labeled, and single-labeled areas were traced and calculated as described.^75,76^ The terminology used is that recommended by the Histomorphometry Nomenclature Committee of the American Society of Bone and Mineral Research.^77^

### Mechanical testing of femurs from LPL^−/−^ and WT mice

Left femur and tibia of 9- and 13-week-old mice were chosen for biomechanical testing. Breaking strength of the left femur was measured under three-point bending using a material testing machine (ElectroForce Systems Group, Bose, Eden Prairie, MN) fitted with a 1 000 N load cell as previously described.^78–80^ The effect of shear loading was minimized via maximizing the distance between the lower support points. Femurs were placed on the loading fixture anterior side down and loaded in the anterior–posterior plane at a span length of (Femur: 9 weeks—9.2 mm, 13 weeks—9.6 mm; Tibia: 9 weeks—13.0 mm, 13 weeks—13.8 mm). Before testing, femora were thawed in saline at room temperature to ensure hydration. Femurs were loaded to failure at a rate of 0.05 mm·s^−1^, during which displacement and force were collected (100 Hz). Force and displacement values were normalized using terms derived from engineering analysis of three-point bending. Bending moments were calculated from the force (*F*) data (*M* = *FL*/4) (N·mm). Displacement data were divided by *L*^2^/12 (mm·mm^−2^), where *L* is the distance between the lower supports (19.26 mm). Whole-bone mechanical properties were then determined from the moment vs. normalized displacement curves, including peak moment (N·mm, ultimate load the specimen sustained), yield moment (N·mm), stiffness (N·mm^2^, the slope of the initial linear portion of the moment–displacement curve), yield displacement (mm·mm^−2^, displacement at the yield point), post-yield displacement (mm·mm^−2^), work to failure (N·mm–mm·mm^−2^, the area under the moment–displacement curve before failure), and work to failure post-yield (N·mm–mm·mm^−2^). The yield point was calculated as the point where a 10% change in the slope of the moment vs. the normalized displacement curve occurred.

### Serum biomarkers

Blood was collected by cardiac puncture in heparin-lithium coated tubes (Thomas Scientific) from WT and LPL^−/−^ mice under deep terminal anesthesia. Blood was collected, and the serum was isolated by centrifugation at 3 000 *g* for 10–15 min. Serum was stored at −80 °C until use. Serum levels of mouse TNF-α, RANKL, carboxyl-terminal telopeptide or carboxyl-terminal collagen cross-link (CTX-1), and tartrate-resistant acid phosphatase 5 isoform b (TRAcP5b) were measured by ELISA kits (R&D Systems, Immunodiagnostics Systems, and Biomedical Technologies) according to the manufacturers’ instructions. Calcium levels were measured using a QuantiChrom Calcium Assay Kit (DICA-500) (BioAssay Systems).

### Statistical analysis

Results are presented as mean ± SD or SEM. Statistical significance was performed using Student’s *t* test (INSTAT; Version 6.0, Graph Pad Software, Graph Pad Inc, San Diego, CA). A probability value <0.05 was considered to be statistically significant.

## Supplementary information

Supplementary Tables and figures
