# Supplementary material for: L-Plastin deficiency produces increased trabecular bone due to attenuation of sealing ring formation and osteoclast dysfunction
Source: Bone Res. 2020 Jan 22;8:3. doi: 10.1038/s41413-019-0079-2 (PMC6976634; doi:10.1038/s41413-019-0079-2)
Supplement: Supplementary file 1 — Supplementary Tables and figures [file 41413_2019_79_MOESM1_ESM.docx]

**Supplementary Tables and Figures**

| **Parameters** | **Tibial histomorphometry**  **(8wks)** | | **Distal femoral histomorphometry (12wks)** | |
| --- | --- | --- | --- | --- |
|  | **WT** | **LPL-** | **WT** | **LPL-** |
| Cancellous Bone (%)  Trabecular thickness (µm)  Trabecular number (#/mm)  Trabecular spacing (µm)  OCs/tissue area (#/mm^2^)  Number of osteoclasts  Eroded perimeters (%)  Number of osteoblasts  Cortical width (µm)  Mineral apposition rate  (µm^2^/µm/day)  Bone formation rate  (µm^2^/µm/day) | 18.8 ± 3  21.9 ± 4  4.3 ± 0.4  162.9 ± 11  136.6 ± 9  218.2 ± 14  6.0 ± 2  211.0 ± 32  196.0 ± 6.7  0.73 ± 0.16  0.53 ±0.11 | 26.8 ± 5*  44.7 ± 6*  6.7 ± 0.6*  124.4 ± 9*  148.0 ± 11  269.0 ± 22  4.0 ± 0.6*  234.0 ± 42  227.0 ± 22  0.66 ± 0.21  0.47 ±0.09 | 38.0 ± 5.2  36.1 ± 4.3  6.9 ± 0.8  136.2 ±12.4  156.0 ± 8.3  223 ± 22  5.86 ± 1.8  220.0 ± 36  247.0 ± 29  0.80 ± 0.14  0.48 ± 0.05 | 51.2 ± 7*  86.5 ±11**  9.0 ±1*  109.1 ± 8*  176.0 ±13  282.0 ± 36  2.5± 1*  284.0 ± 54  298.0 ± 36  1.2 ± 0.3  0.56 ± 0.12 |

**Table S1 Histomorphometry of Bones isolated from Wild-type and LPL-/- mice**

Longitudinal sections of tibial and femoral sections were made and stained with H and E stain or TRAP to detect osteoclasts. Histomorphometric analyses were done using Bioquant-Osteo image analysis software. Analyses were done twice. 5-7 mice were used for each group. Data shown are mean ± SEM; *p<0.05; **p<0.01 vs. WT mice. Standard Student’s t-test assessed P values.

**Table S2: Mechanical strength of left femurs and tibia of 12-week old WT and LPL-/- mice**

Three-point bending tests were conducted in the left femoral and tibial bones of 13-week old WT and LPL-/- mice as described in the Materials and Methods. Mechanical properties were determined from the bending moment vs. normalized displacement curves. P- values indicated in the table are versus WT mice. Differences between the two groups (WT and LPL-/-) were determined by unpaired t-tests (two-tailed) with a significance value set at 0.05.


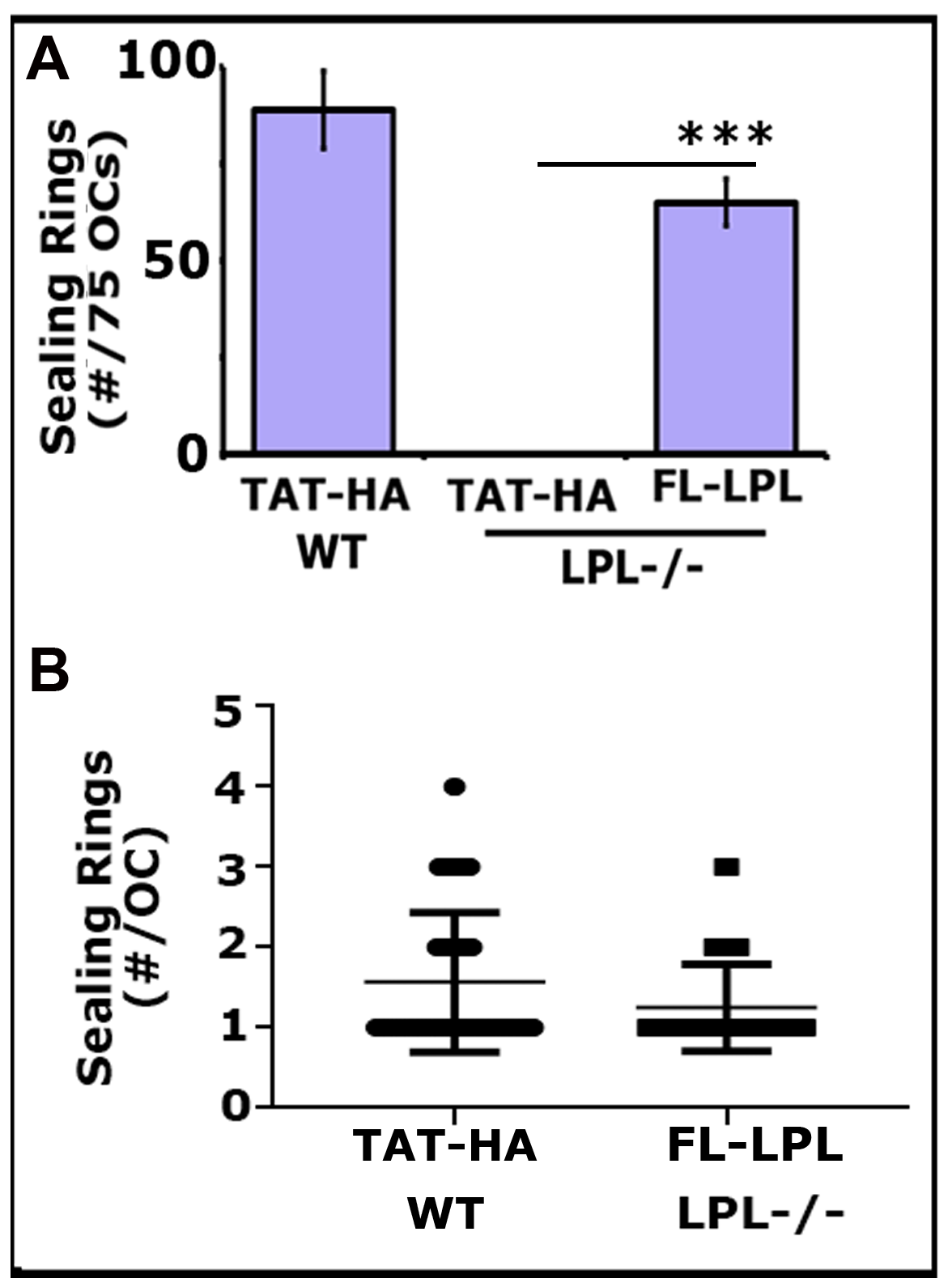


**Fig.S2:** Statistical analysis of the number of sealing rings in WT and LPL-/- osteoclasts transduced with indicated TAT-proteins below the graphs (A and B). A. The total number of sealing rings was counted in ~75 osteoclasts total from three different experiments and presented as a graph. ***p<0.0001 versus TAT-HA protein transduced LPL-/- osteoclasts; error bars indicate mean ± SD. Data shown are for 60 osteoclasts. B. The number of sealing rings per osteoclast is also provided in scatterplot. P-values were assessed by standard Student’s *t*-test


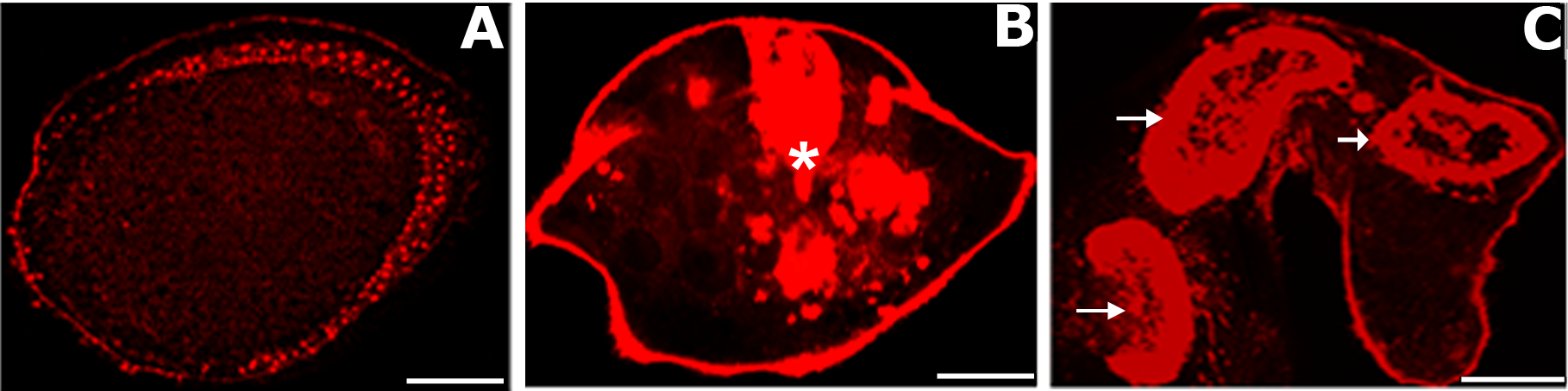


**Fig.S1:** Confocal analysis of actin distribution in osteoclasts plated on glass coverslips (A) and dentine slices (B and C)

Osteoclasts plated on dentine slice for 3-4h (B) and 10-12h (C) as well as plated on glass coverslips (A) in the presence of TNF-α were stained with rhodamine phalloidin to determine the distribution of actin during adhesion (A) and resorption (B and C). NSZs are indicated by an asterisk (B) and mature sealing rings by arrows (C). Scale bar-25μm


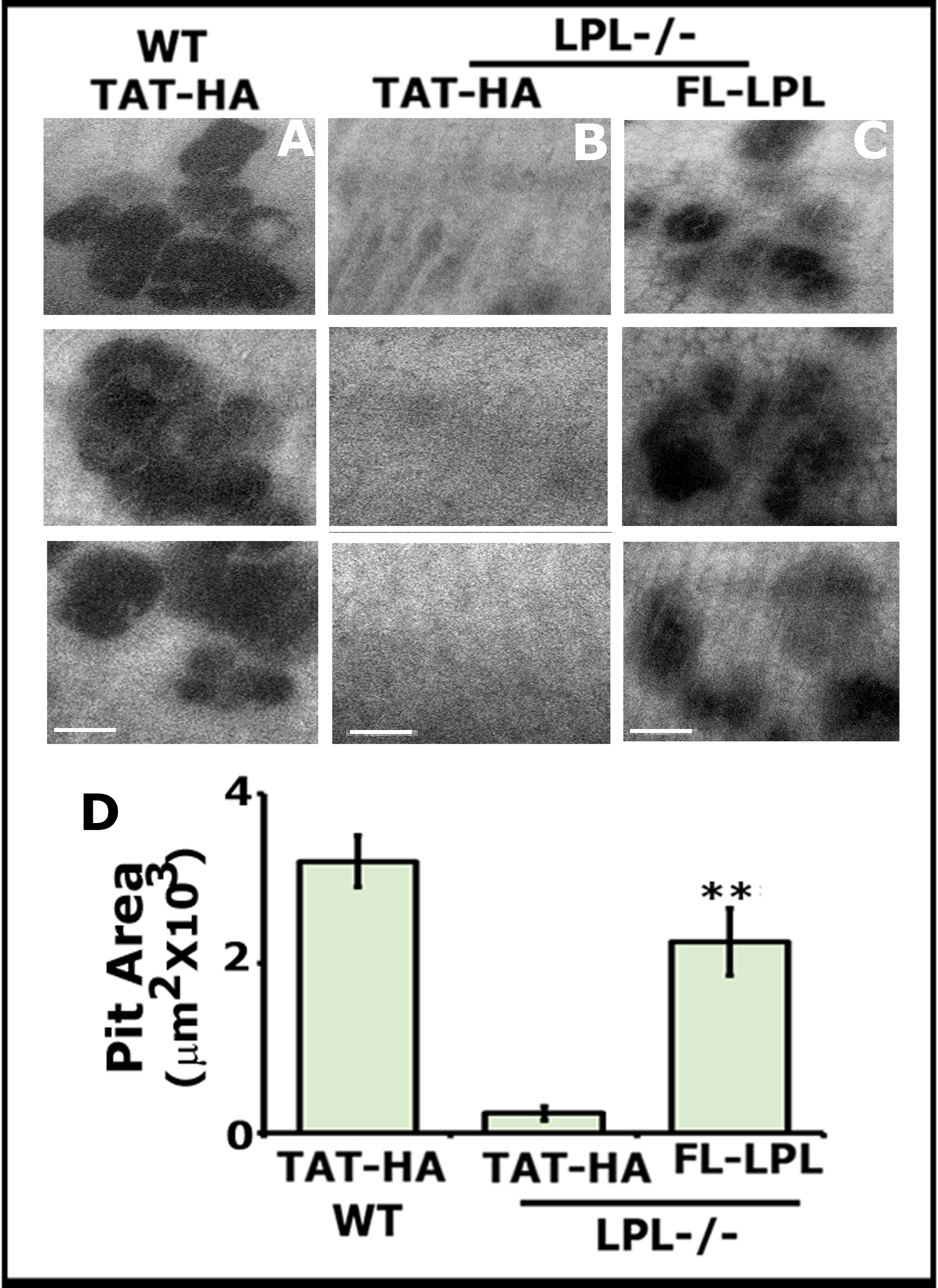


**Fig. S3:** Analysis of resorption activity by osteoclasts using dentine slices. WT and LPL-/- osteoclasts transduced with indicated TAT-fused proteins (Panels A–C) were cultured on dentine slices for 10–12h in the presence of TNF-α. Resorption pits were scanned using a Bio-Rad confocal microscopy. Scale bar- 25µm. Each treatment in the resorption assay was performed in triplicates or quadruplicates and experiments were repeated thrice with three different osteoclast preparations. Images shown in triplicates in A-C are from three different experiments Pit areas were quantiﬁed in three to four slices (6-10 pits/slice) per treatment/experiment and averaged over the three experiments. Statistic measurements for the pit area are provided as a graph in D. **p<0.001 versus TAT-HA transduced LPL-/- osteoclasts; error bars indicate mean ± SD. Analysis was performed using Student's *t-* test


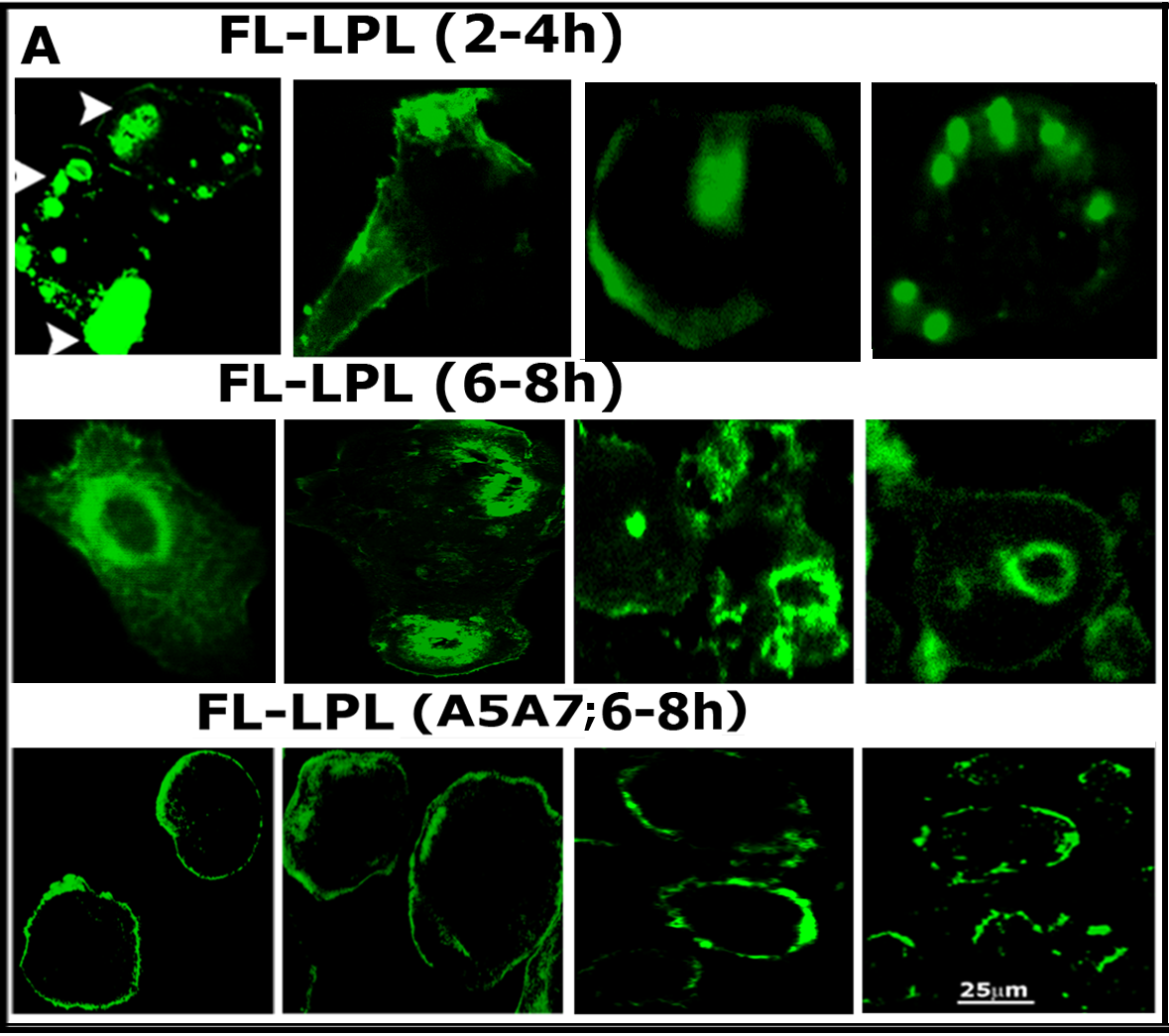


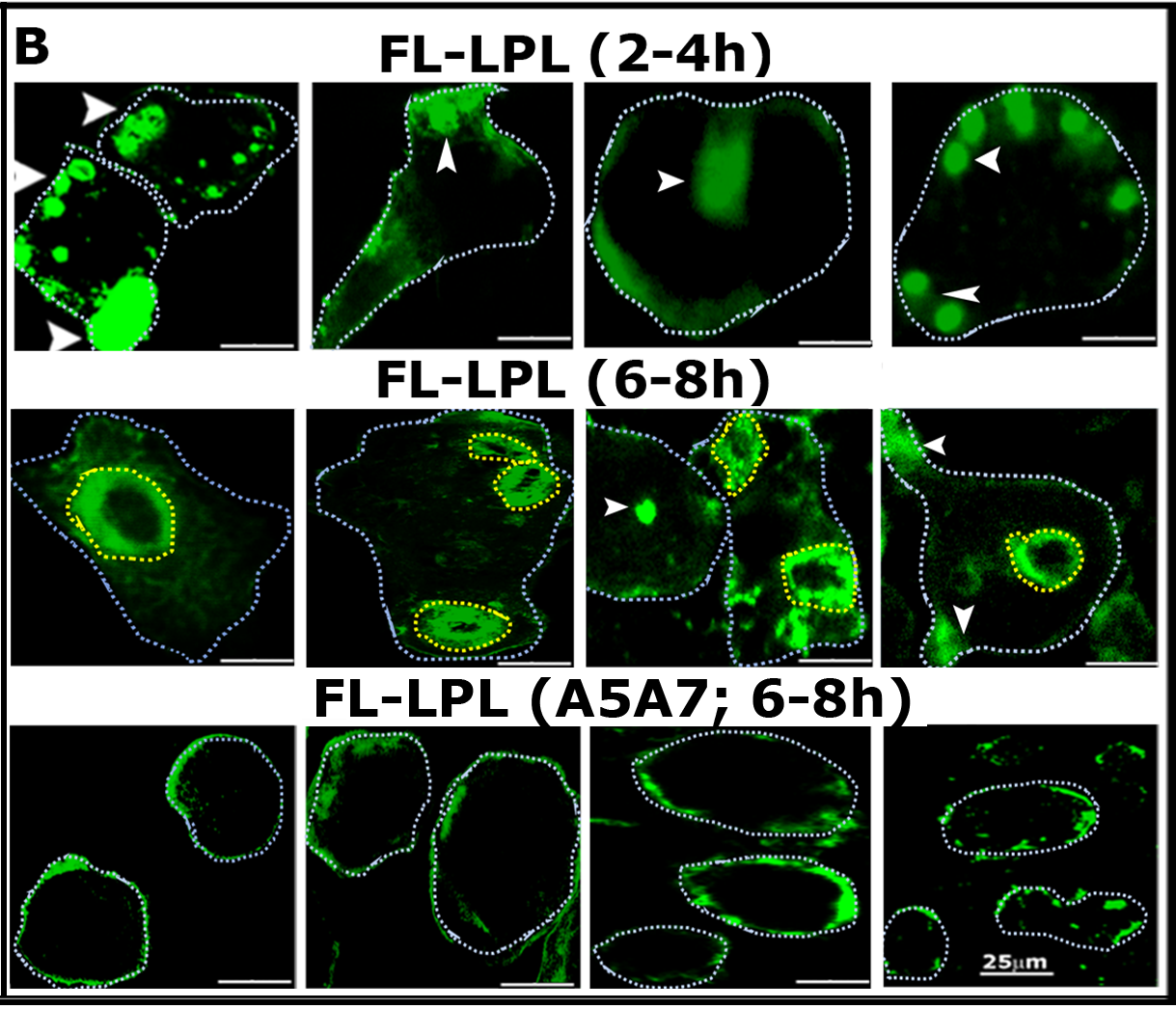


**Fig.S4**: Demonstration of the effects of TAT-fused FL-LPL or FL-LPL (A5A7; panel H) is shown in quadruplicates from three different experiments (A and B). As shown in Figure 5, time lapse video analyses were done at 2-4h (top panels) and 6-8h (middle and bottom panels). NSZs are indicated by arrowheads in B. Single and multiple sealing rings were observed at 6-8h in LPL-/- osteoclasts transduced with FL-LPL. Cells and sealing rings are outlined with light blue and yellow dotted lines, respectively in B. Neither NSZs nor sealing rings were observed in LPL-/- osteoclasts transduced with FL-LPL (A5A7) for 6-8h (bottom panels). Scale bar-25μm


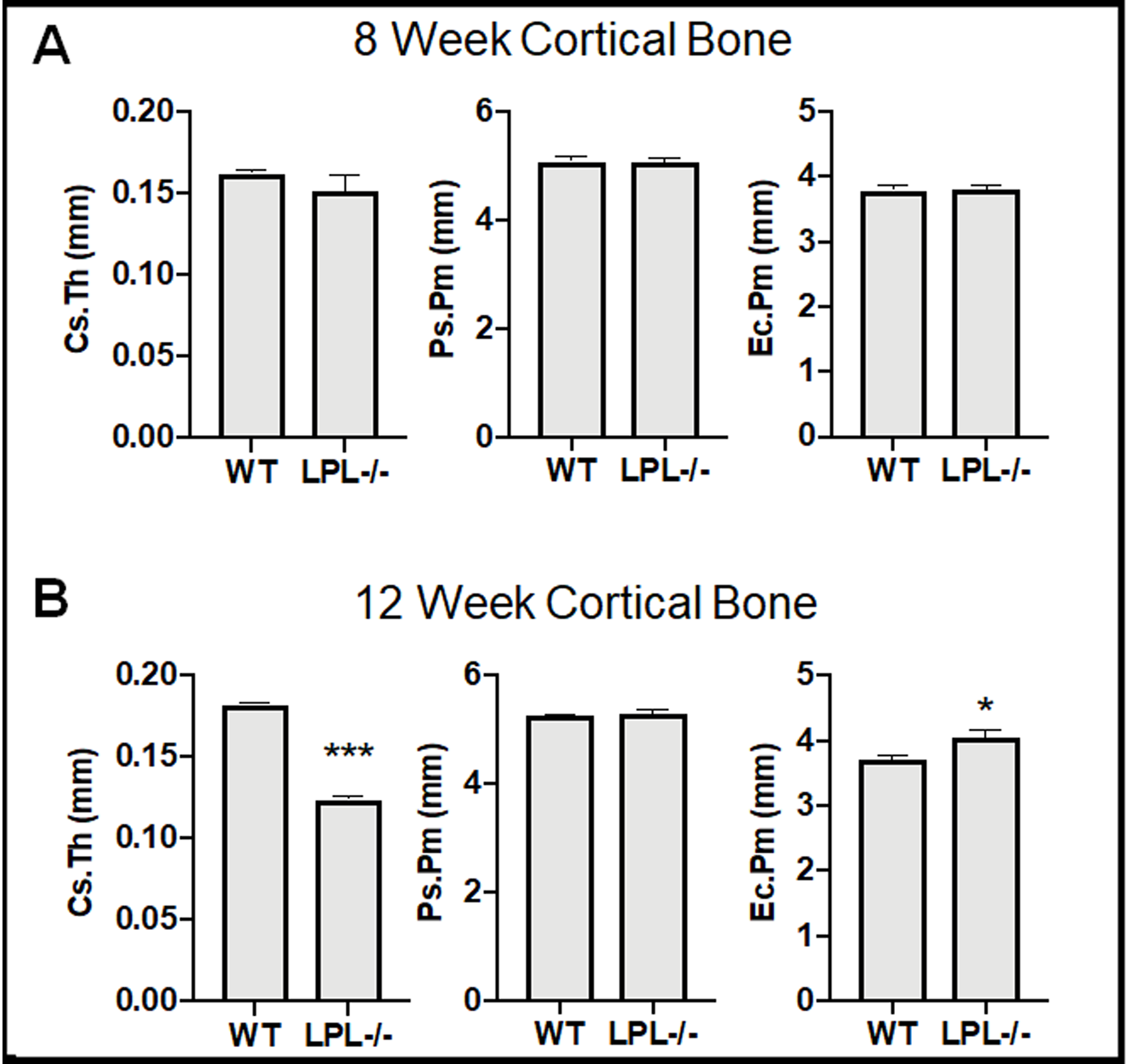


**Fig. S5:** MicroCT analysis of the cortical bone of wild type and LPL-/- mice.

Cortical bone microarchitecture was assessed by micro-CT at the mid-diaphysis of the femur in 8 (A) - or 12-week (B) old WT or LPL-/- female mice by micro-CT (n=5-6/group). Cortical cross-sectional thickness (Cs.Th), periosteal perimeter (Ps.Pm), and endocortical perimeter (Ec.Pm) are shown. Data are mean ± SD. *p<0.05; ***p<0.001 vs. WT mice. Analyses were performed using Student's *t-* test


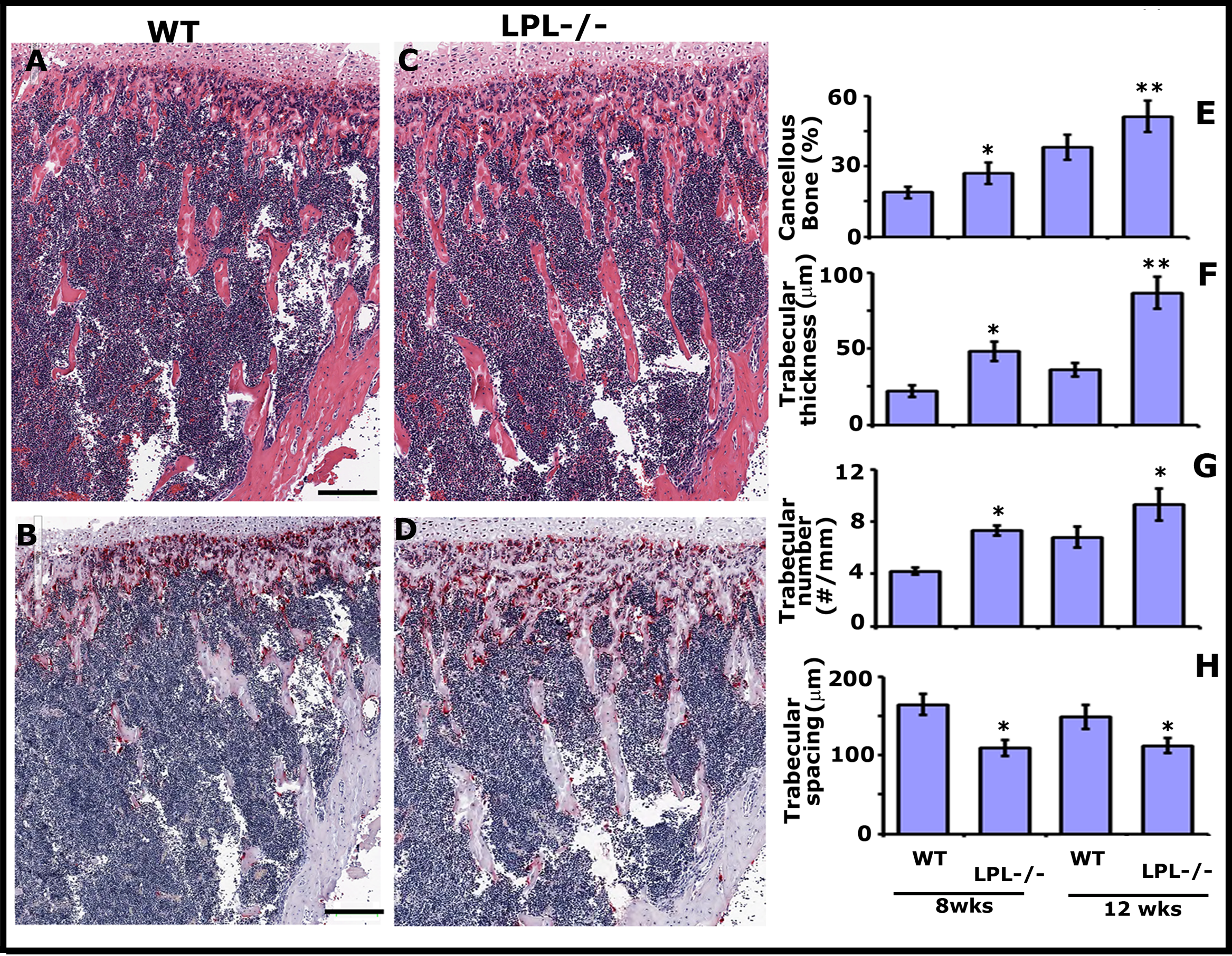


**Fig. S6:** Histological and histomorphometric assessment of bone properties in the proximal tibial sections of wild-type (WT) and LPL-/- mice.

Histological sections of the proximal tibial bone sections of 8-week old mice stained with H and E (A and C) or TRAP (B and D) are shown. Histomorphometry analyses were done in 8 and 12 week old mice and value for the analyzed parameters are provided as a table (Table S1). Graphs were plotted for parameters which show a considerable change in LPL-/- mice (panels E-I). Data are mean ± SEM. *p<0.05; **p<0.01 vs. WT mice. Scale bar- 300µm. Standard Student’s t-test assessed P values.


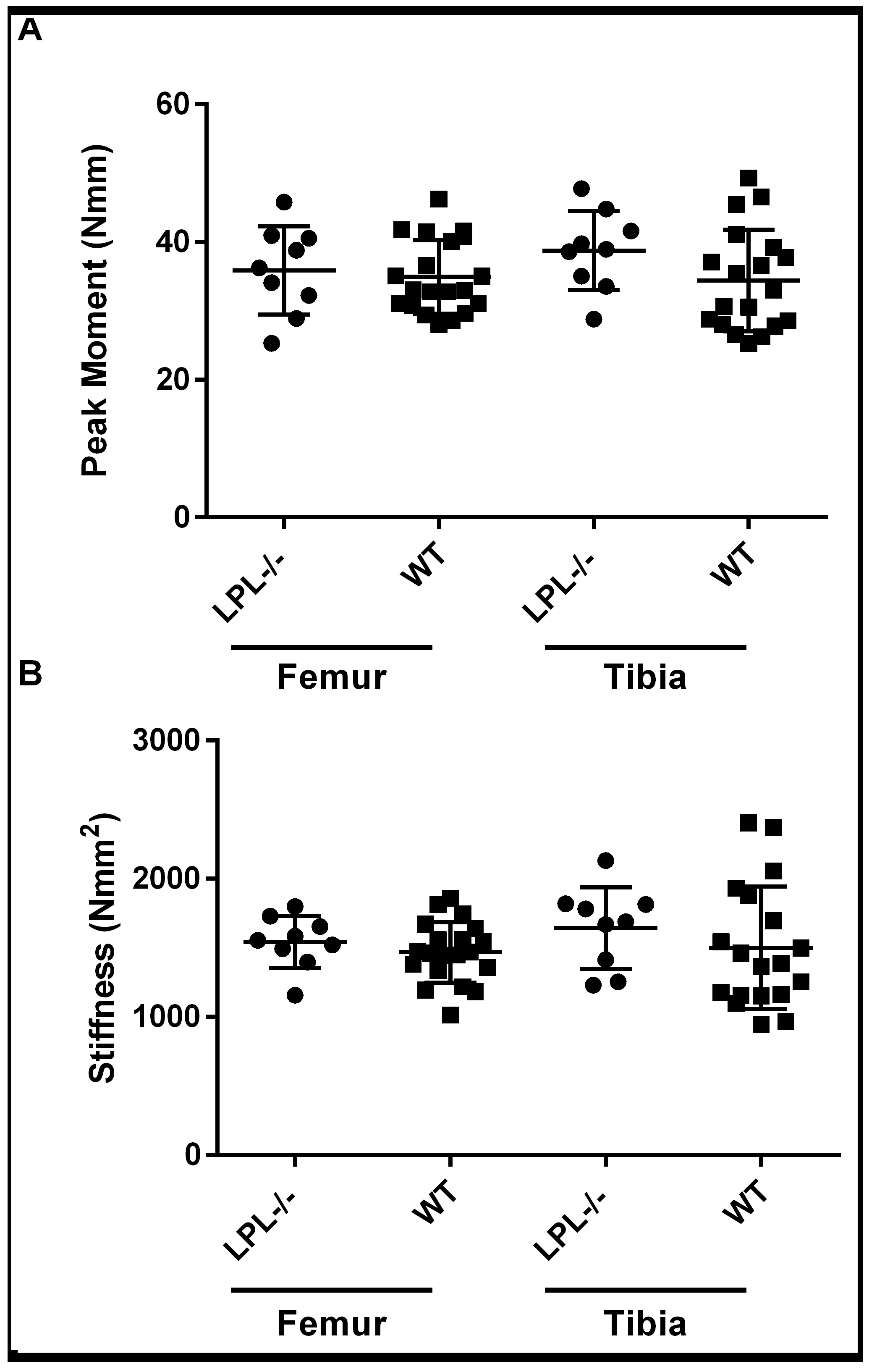


**Fig. S7:** Mechanical studies in the left femoral and tibial bones of 13 weeks old WT and LPL-/- mice

Table S2 provides the value for the parameters investigated. Data shown are mean ± SD of indicated mice in the table. Data are also given as scatterplots in A and B for the peak moment and stiffness for the indicated number of mice in Table S2. All statistical analyses were performed using Graph Pad (Graph Pad Prism version 8.00 for Windows, Graph Pad Software, San Diego, CA, USA). Differences between groups (WT and LPL-/-) were determined using unpaired t-tests (two-tailed) with a significance value set at 0.05.
